# Effects of dietary sodium butyrate on growth performance, immune function, and intestinal microflora of Chinese soft-shelled turtle (*Pelodiscus sinensis*)

**DOI:** 10.3389/fcimb.2023.1271912

**Published:** 2023-10-11

**Authors:** Lingrui Ge, Yi Yu, Xingxing Wen, Hewei Xiao, Kejun Liu, Zhuying Liu, Shuai Liu, Qian Li, Xiaoqing Wang, Zaofu Deng, Yazhou Hu

**Affiliations:** ^1^ College of Fisheries, Hunan Agricultural University, Changsha, China; ^2^ College of Animal Science and Technology, Hunan Biological and Electromechanical Polytechnic, Changsha, China

**Keywords:** aquatic feed, sodium butyrate, *Pelodiscus sinensis*, immunity, intestinal microbe

## Abstract

The Chinese soft-shelled turtle (*Pelodiscus sinensis*) has become increasingly susceptible to frequent diseases with the intensification of farming, which severely impacts the development of the aquaculture industry. Sodium butyrate (SB) is widely used as a feed additive due to its promotion of growth, enhancement of immune function, and antioxidative properties. This study aimed to investigate the effects of dietary SB on the growth performance, immune function, and intestinal microflora of Chinese soft-shelled turtles. A total of 300 Chinese soft-shelled turtles (mean weight: 11.36 ± 0.21g) were randomly divided into four groups with three parallel sets in each group. Each group was fed a diet supplemented with 0%, 0.005%, 0.01%, or 0.02% SB for 60 days. The results demonstrated an upward trend in weight gain rate (WGR) and specific growth rate (SGR) with increasing SB supplementation, and the experimental group fed with 0.02% SB showed a significant increase in WGR and SGR compared to other groups (*P*< 0.05). These levels of SB also decreased the levels of feed conversion ratio (FCR) and the total cholesterol (TC) content of Chinese soft-shelled turtles, and the 0.02% SB was significantly lower than that of other groups (*P*< 0.05). The activity of complement protein *in vivo* increased with increases in SB content, and the activities of complement C3 and C4 reached the highest level with 0.02% SB. The species abundance of the experimental group D fed with 0.02% SB was significantly higher than that of other groups (*P<* 0.05). Furthermore, the relative abundance of *Clostridium sensu stricto* 1 was significantly increased with 0.02% SB (*P*< 0.05). In conclusion, adding 0.02% SB to the diet improves the growth performance, feed digestion ability, and intestinal microbiota of Chinese soft-shelled turtles.

## 1 Introduction

The Chinese soft-shelled turtle (*Pelodiscus sinensis*) is a benthic aquatic animal that inhabits freshwater environments. Due to its highly sought-after nutritional value, it occupies a prominent position in traditional aquaculture in China. Renowned for its richness in protein and low-fat content, it remains a popular choice in the market. Among its various components, the skirt edge stands out with the highest crude protein content. Furthermore, the muscle of the Chinese soft-shelled turtle boasts an essential amino acid index exceeding 90%, making it a highly nutritious option (Huang and Bao, 2023). Many ponds in China cultivate Chinese soft-shelled turtles to meet market demands. However, with the intensification and refinement of cultivation, high breeding densities and insufficient carrying capacity in the ponds disrupt the aquatic animals’ mechanisms and bacterial balance (Yarahmadi et al., 2015), resulting in the proliferation of bacterial pathogens and subsequent deaths of Chinese soft-shelled turtles due to bacterial diseases (Chung et al., 2017). Previous studies have demonstrated that too many fish raised in a pond beyond its environmental capacity could trigger diseases and cause environmental pollution (Tang et al., 2021). In such intensive environments, aquatic animals reduce their feed intake and intestinal digestive capacity. To enhance the growth and immune ability of aquatic animals, numerous studies have been conducted on feed additives.

At present, SB is widely used as a feed additive due to its ability to promote growth and improve immune function and antioxidant properties (Abdel-Latif et al., 2020). Studies have demonstrated that SB can provide a fast and easily absorbed energy source for animals and exhibit a diverse array of biological effects (Cummings and Macfarlane, 1997). Furthermore, some researchers found that SB can repair the intestinal epidermal mucosa, which has protective effects on the intestinal tract of aquatic animals (Du et al., 2023). The profound impact of SB on the health of aquatic animals has led to its widespread adoption as a feed additive. (Tran et al., 2020). Although there are reports on the effects of SB supplementation in fish diets, studies on its supplementation in turtle diets are required. Hence, this study aimed to investigate the effects of SB on the growth performance, immune function, and gut microbiota of Chinese soft-shelled turtles.

## 2 Materials and methods

### 2.1 Experimental feed design

**Table 1** shows the nutritional composition of the basic feed. The protein source of the experimental feed was imported fish meal, soybean meal, and other raw materials, and the fat source was fish oil, peanut oil, and other raw materials. Four treatment diets were prepared by adding 0%, 0.005%, 0.01%, or 0.02% SB to the basic diet. The raw materials were crushed using an 80-mesh screen to remove large particles, and then precisely weighed according to the proportion of the feed formula. The mixed feed was put into a batch mixer, and a fixed amount of distilled water was added for full mixing. Then, the mixed feed was made into particles with a 1.5-mm diameter using a ring mold granulating machine and dried in an oven at 60 °C. Finally, the feed was air-dried and collected in sealed bags, which were stored in a freezer at −25 °C.

**Table 1:** Nutrient composition and nutrient level of basic feed.

Nutritional composition	Content (%)	Nutrient levels ②	Content (%)
Imported fishmeal	32.00	Crude protein (CP)	30.52
Gelatin	9.00	Crude fat (EE)	4.35
Soybean meal	9.00	Coarse ash (Ash)	14.76
Corn gluten meal	9.00	Moisture	11.85
Corn starch	16.00		
Fish oil	2.50		
Peanut oil	2.45		
premix ①	3.30		
Choline chloride	0.15		
Ascorbic acid	0.05		
Sodium carboxymethylcellulose	2.00		
Microcrystalline cellulose	14.55		
Total	100		

① Add premix according to the requirements of feed per kilogram. Vitamin content in premix: Vit A 6000 IU, Vit B1 6 mg, Vit B2 15 mg, Vit B6 8 mg, Vit C 8 mg, Vit D 2000 IU, Vit E 100 IU, folic acid 7 mg, biotin 0.3 mg; Mineral content: Se 0.5 mg, I 1 mg, Cu 5 mg, Mn 20 mg, Zn 65 mg, Fe 70 mg. ② Nutrient levels were measured by instruments in our laboratory.

### 2.2 Husbandry methods

The 300 healthy Chinese soft-shelled turtles (mean weight: 11.36 ± 0.21 g) were obtained from Changde Hezhou Aquatic Products Co., Ltd, Changde, China. All animal studies were performed according to protocols approved by the Animal Care Advisory Committee of Animal Science and Technology, Hunan Agricultural University. They were randomly divided into four groups: the control group (group A) was fed experimental diets without SB, while the experimental groups B, C, and D were fed experimental diets supplemented with 0.005%, 0.01%, and 0.02% SB (Hangzhou Kantian Biotechnology Co., Ltd, Hangzhou, China), respectively. The Chinese soft-shelled turtles were raised in an indoor glass tank (1 m × 0.5 m × 0.5 m, length × width × depth) with water exchanged once every 2 weeks to ensure optimal growth conditions. To control the variables, the source of water change during feeding was purchased pure water, and the water temperature in the glass tank was kept at 25 °C~32 °C. Before the trial, Chinese soft-shelled turtles were starved for 2 days. At the beginning of the experiment, the body weight of the Chinese soft-shelled turtle was recorded, and they were fed twice a day with a prescribed dosage of 2% of their body mass. The feeding quantity was adjusted by weighing the turtles every 2 weeks. The remaining feed was collected one hour after each feeding to avoid its influence on water quality. The growth performance of each group was tested at 30 d and 60 d. At 60 d, 7 Chinese soft-shelled turtles were randomly selected from each group, and serum immune indexes and intestinal microbial population data were collected. The control group (group A) consisted of Chinese soft-shelled turtles labeled A1–A7, while the experimental groups B, C, and D consisted of Chinese soft-shelled turtles labeled B1–B6, C1–C7, and D1–D7, respectively.

### 2.3 Measurement and calculation of growth performance

Each group of Chinese soft-shelled turtle individuals was weighed on day 30 and day 60 of the experiment, and the body weight gain, body length, and number of deaths of Chinese soft-shelled turtle were recorded. Their daily food intake was calculated, and their liver mass was weighed. The following formulas were used to calculate their growth performance:


(1) $$\text{Weight gain (WG)}={\text{W}}_{\text{t}}-{\text{W}}_{\text{O}}$$

(2) $$\text{Specific growth rate }(\text{SGR})=({\text{lnW}}_{\text{t}}-{\text{lnW}}_{0})/\text{t}\times 100\%$$

(3) $$\text{Feed conversion ratio (FCR)}=\text{WTC}/\left[{\text{(W}}_{\text{t}}-{\text{W}}_{0})\times \text{n}\right]$$

(4) $$\text{Hepato-somatic index (HSI)}={\text{W}}_{\text{h}}/{\text{W}}_{\text{t}}\times 100$$

(5) $$\text{Survival rate (SR)}={\text{N}}_{\text{t}}/{\text{N}}_{0}\times 100\%$$

The variables used in the calculations were as follows: Wt represents the body mass of Chinese soft-shelled turtles at 30 d and 60 d; W_0_ stands for their initial mass; t represents the number of days of testing; WTC stands for the total food intake during the trial phase; n represents the number of turtles in each group; W_h_ stands for their liver mass; N_t_ stands for the final number of turtles; and N_0_ represents their initial number.

### 2.4 Determination of serum indexes

After feeding, 7 Chinese soft-shelled turtles were randomly selected from each group for blood collection. The collected blood was placed in a refrigerator at 4 °C for 4 h, followed by centrifugation for 10 min at 4 °C and 5000 RPM. After centrifugation, the upper serum was collected and serum indexes were detected using the kit from Nanjing Jiancheng Bioengineering Research Institute. The serum biochemical indexes included TC, triglyceride (TG), total protein (TP), aspartate aminotransferase (AST), and alanine aminotransferase (ALT). Serum immune indices were superoxide dismutase (SOD), malondialdehyde (MDA), and complement C3 and C4.

### 2.5 Gut microbial changes and bioinformatics analysis

#### 2.5.1 Intestinal sample collection

To study the intestinal microbial population of Chinese soft-shelled turtles, we sampled the intestinal tract of experimental and control groups. Before sampling, we anesthetized the turtles by placing them in water with MS-222 (100 μg/L) for 2 min. We then wiped 75% ethanol on the surface of the turtles, quickly disassembled them, removed their intestine, and squeezed the intestinal contents into a 5-ml centrifuge tube. The samples were then frozen with liquid nitrogen and stored at −80 °Cfor analysis of intestinal flora changes.

#### 2.5.2 Analysis of microflora changes in intestinal samples

The intestinal genome DNA of Chinese soft-shelled turtles was extracted and sequenced using the combined multiomics technology of Shanghai Oyi Biotics. Genomic DNA was extracted from the sample with a DNA extraction kit, and the DNA concentration was detected using agarose gel electrophoresis and a NanoDrop spectrophotometer. The extracted genomic DNA was used as a template to delimit the genomic sequencing area, and specific primers were added to the DNA template, followed by mixing. The mixture was then put into a PCR instrument (Takara) for testing, and the accuracy and validity of the detection value were guaranteed.

The raw data obtained were in FASTQ format, and Cutadapt software was used to process the raw sequence to cut off the primer sequence. After that, DADA2 was used to process the original sequence according to Qiime2 setting procedures to obtain high-quality sequences and amplicon sequence variants (ASV) abundance tables. The diversity of the obtained data was analyzed, and the intestinal microflora information of Chinese soft-shelled turtles was obtained through various algorithms. This information was used to analyze SB-driven changes in the intestinal microflora of Chinese soft-shelled turtles.

### 2.6 Statistical analysis

Statistical analysis was performed according to Excel to sort out the recorded data. The recorded data results were expressed as Avg ± SD. The normality of the data was tested by SPSSAU, and then Duncan was used to conduct one-way ANOVA on the obtained data to identify significant differences (*P<* 0.05).

## 3 Results

### 3.1 Changes in growth performance

According to **Tables 2**, **3**, the WGR and SGR of Chinese soft-shelled turtles supplemented with SB showed an upward trend with increases in SB concentration. WGR and SGR values in group D (0.02% SB) were significantly higher than those in the other groups (*P*< 0.05). The FCR showed a decreasing trend with increases in SB concentration. FCR values in group D (0.02% SB) were significantly lower than those in the other groups (*P<* 0.05). Although the HSI of each group fluctuated, the overall difference was not significant (*P* > 0.05).

**Table 2:** Growth performance of Chinese soft-shelled turtle on day 30.

Items	Control group (A)	Experimental group(B)	Experimental group(C)	Experimental group(D)
IBW/g	11.35±0.13	11.44±0.12	11.39±0.18	11.26±0.11
BW of day 30/g	17.12±0.56^a^	18.85±0.93^ab^	18.46±0.75^ab^	20.21±0.41^b^
WGR/g	5.77±0.60^a^	7.41±0.04^ab^	7.07±0.11^ab^	8.95±0.90^b^
SGR/(%/d)	1.43±0.05^a^	1.71±0.09^ab^	1.68±0.06^ab^	1.92±0.04^b^
FCR (%)	1.22±0.13^b^	1.16±0.09^b^	0.94±0.15^ab^	0.86±0.08^a^

In the data, letters are used to indicate significant differences in the data of the group (P< 0.05), and the same or no letters are used to indicate no significant differences in the data of the group (P > 0.05). IBW, Initial body weight; BW, body weight; WGR, weight gain rate; SGR, specific growth rate; FCR, feed conversion ratio. There is no significant difference between a and ab. There are significant differences between a and b.

**Table 3:** Growth performance of Chinese soft-shelled turtle on day 60.

Items	Control group (A)	Experimental group (B)	Experimental group (C)	Experimental group (D)
IBW/g	11.35±0.13	11.44±0.12	11.39±0.18	11.26±0.11
FBW/g	30.62±0.85^a^	33.54±0.90^ab^	32.45±0.34^ab^	36.31±0.45^b^
WGR/g	19.27±0.30^a^	22.10±0.26^ab^	21.06±0.42^a^	25.05±0.17^b^
SGR/(%/d)	1.65±0.06^a^	1.76±0.10^ab^	1.74±0.06^ab^	1.93±0.08^b^
FCR (%)	1.19±0.15^b^	0.94±0.14^ab^	0.96±0.10^ab^	0.87±0.08^a^
HSI (%)	3.26±0.13	3.18±0.16	3.08±0.24	3.22±0.10

In the data, letters are used to indicate significant differences in the data of the group (P< 0.05), and the same or no letters are used to indicate no significant differences in the data of the group (P > 0.05). IBW, Initial body weight; FBW, final body weight; WGR, weight gain rate; SGR, specific growth rate; FCR, feed conversion ratio; HSI, hepato-somatic index. There is no significant difference between a and ab. There are significant differences between a and b.

### 3.2 Changes in serum biochemical indexes

As shown in **Table 4**, the TC content of Chinese soft-shelled turtles showed a decreasing trend with increased SB concentration. The TC content in group D (0.02% SB) was significantly different from that of other groups (*P*< 0.05). There were no significant differences in other indexes (*P* > 0.05).

**Table 4:** Serum biochemical indexes of Chinese soft-shelled turtle.

Projects	SB addition level			
	0 (Control group)	0.005%	0.01%	0.02%
TP/(g/L)	32.52±1.12	32.12±1.38	29.93±2.59	30.46±1.96
TC/(mmol/L)	6.92±0.24^b^	5.96±0.16^ab^	5.43±0.06^ab^	5.02±0.10^a^
TG/(mmol/L)	1.05±0.25	1.12±0.12	0.96±0.10	0.92±0.08
GPT/(U/L)	20.25±0.65	19.35±0.25	19.62±0.18	20.56±0.75
GOT/(U/L)	104.26±10.36	103.35±10.86	109.65±9.67	112.21±9.21

There is no significant difference between “a” and “ab”. There are significant differences between “a” and “b”.

### 3.3 Changes in serum nonspecific immune indexes

According to **Table 5**, changes in indicators in the serum of Chinese soft-shelled turtles can affect their immune ability. It was found that the activity of complement protein increased with increases in SB concentrations, and the activities of C3 and C4 peaked at 0.02% SB, being significantly different from those of other groups (*P<* 0.05). In the serum immune index data, SOD and malondialdehyde showed a dynamic equilibrium state, indicating that SB had no significant effects on them (*P* > 0.05).

**Table 5:** Serum nonspecific immune indicators of Chinese soft-shelled turtle.

Projects	SB addition level			
	0 (Control group)	0.005%	0.01%	0.02%
SOD/(U/L)	46.71±3.72	48.25±2.58	45.36±3.27	49.12±1.75
MDA(mmol/L)	5.50±0.35	5.37±0.45	5.62±0.27	5.75±0.18
C3/(mmol/L)	0.27±0.02^a^	0.30±0.07^ab^	0.34±0.05^ab^	0.38±0.06^b^
C4/(mmol/L)	0.28±0.05^a^	0.31±0.03^ab^	0.33±0.08^ab^	0.39±0.09^b^

There is no significant difference between “a” and “ab”. There are significant differences between “a” and “b”.

### 3.4 Changes in intestinal flora

#### 3.4.1 Intestinal sample sequencing data

The intestinal sample of Chinese soft-shelled turtles was used to obtain 2,247,884 original sequences from 27 samples by high-throughput sequencing. The primers in the original sequences were cut by Cutadapt, and qualified original sequences were input into Qiime2 after quality filtering, noise reduction, splicing, and dechimerism. A total of 1,844,376 effective high-quality sequences were obtained, with an average effective data ratio of 82%. In DADA2, noise reduction, chimera removal, and duplication removal were performed according to the set procedures, and the resulting sequences were called ASVs. The number of tags assigned to ASVs in each sample was counted separately to determine the abundance of each ASV in each sample. The ASVs of groups A, B, C, and D were 1186, 1208, 1114, and 1300, respectively. As shown in **Table 6**, intestinal sample sequencing data and ASV abundance statistics were used to analyze the intestinal flora diversity of Chinese soft-shelled turtles.

**Table 6:** Intestinal sample sequencing data and ASV abundance statistics.

Projects	Original sequence	High-quality sequence	Valid data ratio/%	ASVs
Control group (A)	561,026	460,617	82.10	1186
Experimental group (B)	562,692	465,554	82.73	1208
Experimental group (C)	566,045	461,271	81.49	1114
Experimental group (D)	558,121	456,934	81.87	1300

#### 3.4.2 Analysis of intestinal flora changes

**Figure 1:**
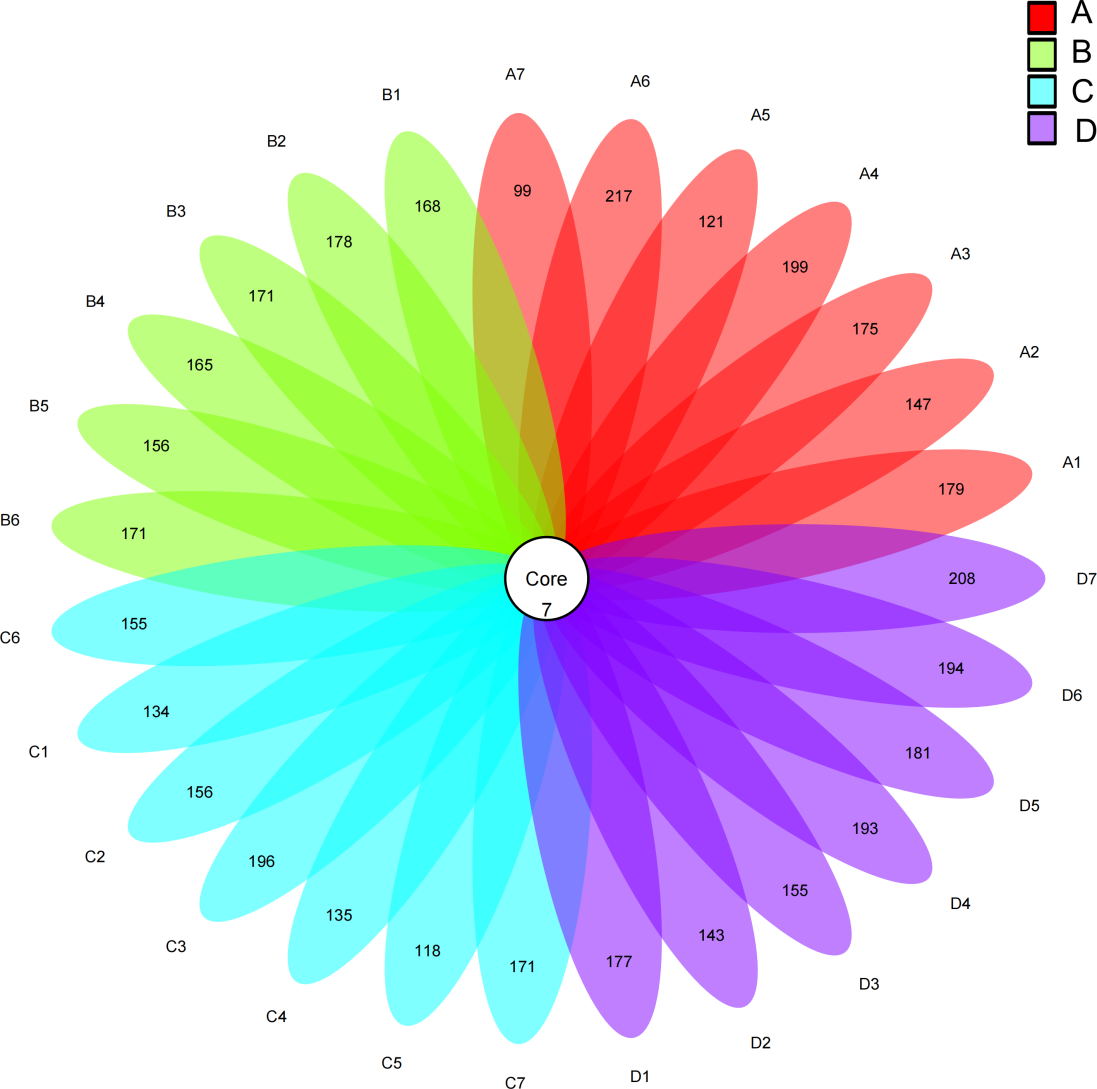
Intestinal flora of Chinese soft-shelled turtle. A1-A7: Control group; B1-B6: Experimental group (0.005% SB); C1-C7: Experimental group (0.01% SB); D1-D7: Experimental group (0.02% SB).

Based on **Figure 1**, 7 ASVs were shared by both experimental and control groups. The numbers displayed in the petal diagram indicated the total ASVs subtracted by the number of common ASVs in each sample, and the unique ASVs of groups A, B, C, and D were 1179, 1201, 1107, and 1293, respectively. Moreover, the species abundance of group D (0.02% SB) was found to be significantly higher than that of other groups (*P<* 0.05).

**Figure 2:**
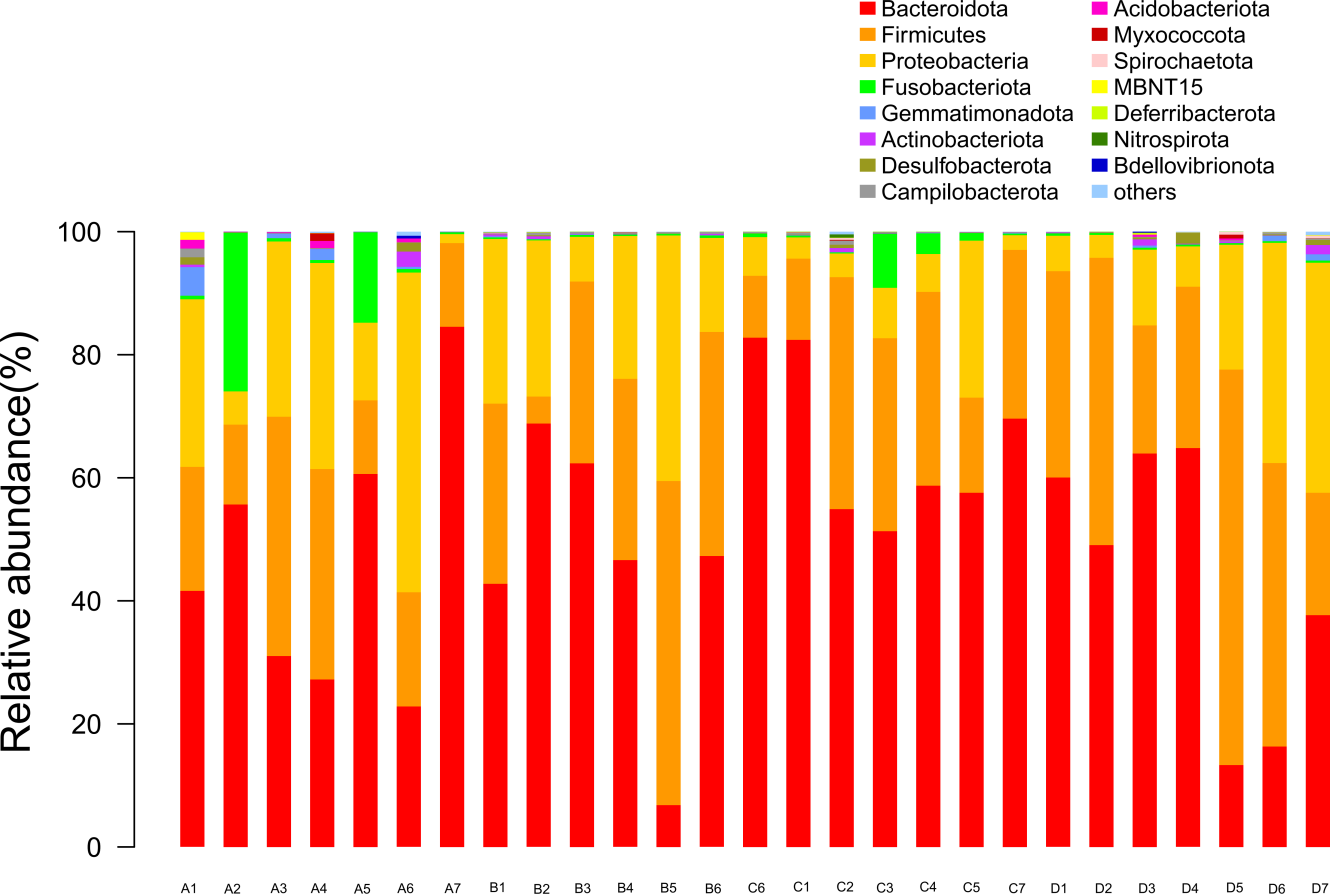
The top 15 bacteria in the intestinal phylum classification level of species abundance. A1-A7: Control group; B1-B6: Experimental group (0.005% SB); C1-C7: Experimental group (0.01% SB); D1-D7: Experimental group (0.02% SB).

Based on the diversity analysis of the top 15 species abundance in the phylum classification level of intestinal bacteria, as illustrated in **Figure 2**, *Bacteroidota*, *Firmicutes*, and *Proteobacteria* were found to be the dominant bacteria present in the intestinal flora of the 4 groups of Chinese soft-shelled turtles. Among them, *Bacteroidota* accounted for 50.3% in the top 15 species abundance of phylum, while the other two *Bacteroidota* accounted for 28% and 17.6%, respectively.Furthermore, the relative abundances of *Bacteroidota* and *Campilobacterota* in experimental group D were significantly higher compared to those of the control group (*P<* 0.05), with the relative abundance of *Campilobacterota* showing a rising trend with increases in SB. However, the relative abundance of *Bacteroidota* in experimental group C was significantly higher than that of other groups (*P<* 0.05), with an SB efficiency rate of 41.34%. Meanwhile, the relative abundances of *Proteobacteria*, *Actinobacteriota*, and *Desulfobacterota* in the experimental groups notably decreased in contrast to those of the control group (*P*< 0.05), and they exhibited a declining trend with increases in SB.

**Figure 3:**
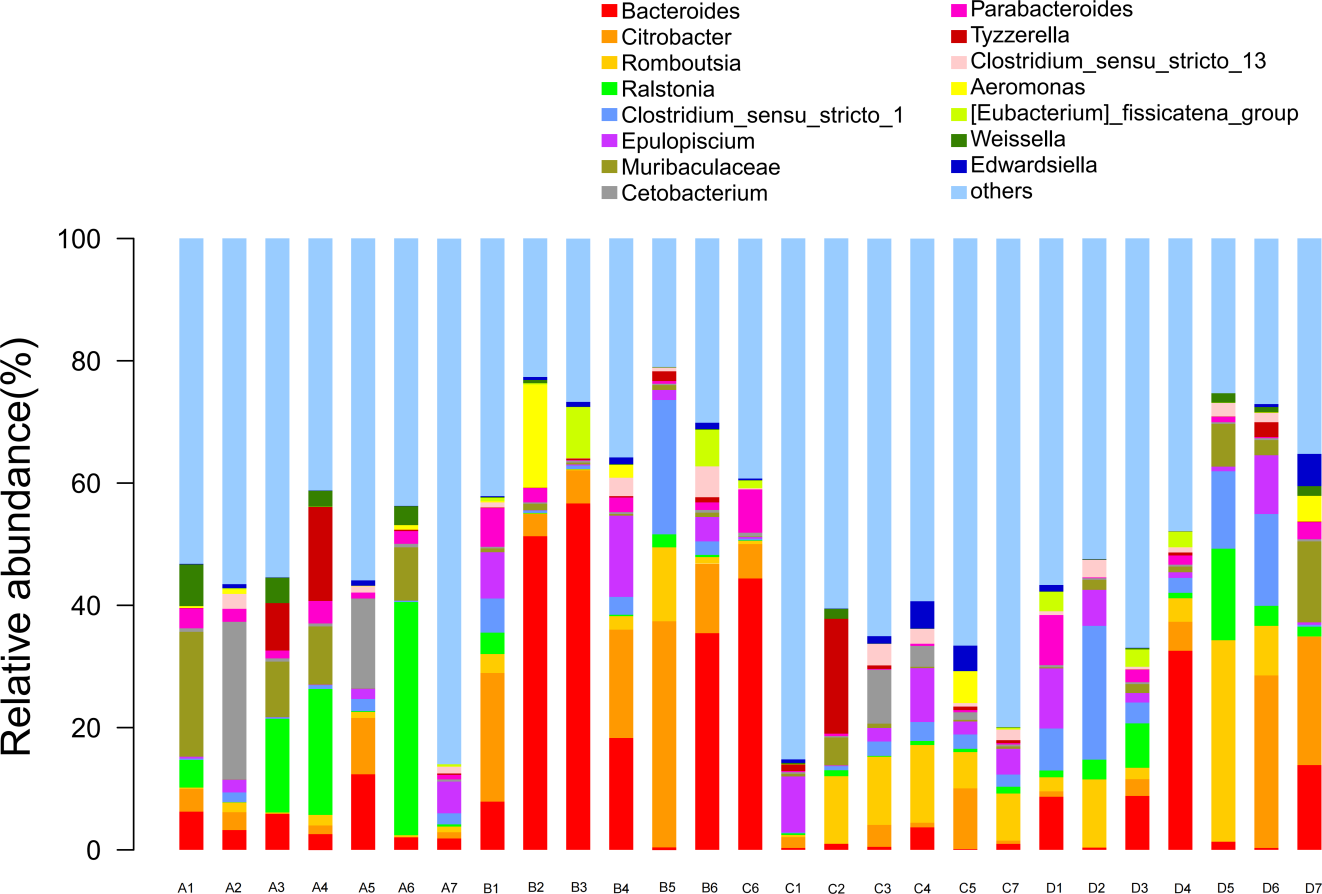
The top 15 bacteria in the intestinal genus classification level of species abundance. A1-A7: Control group; B1-B6: Experimental group (0.005% SB); C1-C7: Experimental group (0.01% SB); D1-D7: Experimental group (0.02% SB).

The diversity analysis of the top 15 species abundance in the genus classification level of intestinal bacteria is depicted in **Figure 3**. *Bacteroides* and *Citrobacter* were identified as the main dominant bacterial genera present in the intestinal flora of the four groups of Chinese soft-shelled turtles, respectively accounting for 11.9% and 7.19% of the top 15 species abundance at the classification level.

Moreover, the relative abundance of *Bacteroides* and *Citrobacter* in all experimental groups was significantly higher than that of the control group (*P*< 0.05). Interestingly, in contrast to the other conditions, the content of SB in the two bacterial groups exhibited an inverse proportion to the species abundance, with a downward trend observed. Additionally, the relative abundance of *Ralstonia* and *Weissella* in the experimental group significantly decreased compared to that in the control group (*P<* 0.05), showing a curve decline. Furthermore, the total species abundance in group C (0.01% SB) was the lowest.

**Figure 4:**
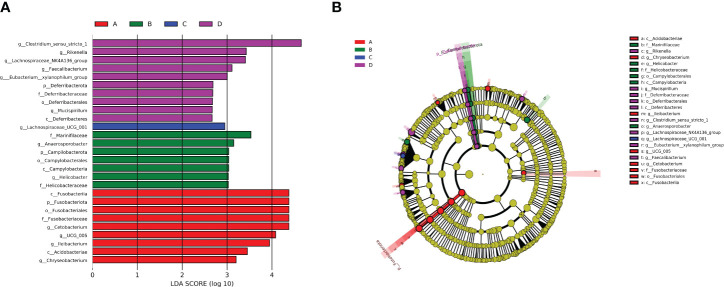
**(A)** LEfSe analysis of intestinal flora. **(B)** Evolutionary branches of phylogenetic relationships. A: Control group; B: Experimental group (0.005% SB); C: Experimental group (0.01% SB); D: Experimental group (0.02% SB).

LefSe analysis is a useful tool for identifying microbial communities that exhibit significant differences in sample distribution. LDA score is typically utilized to determine the impact of a certain microbiome on the abundance, with a judgment criterion of LDA≥2. As shown in **Figure 4A**, 9 significant bacterial genera were identified in the control group, with *Cetobacterium* holding the LDA score of 4.37. On the other hand, 18 bacterial genera played an important role in the experimental group, with *Clostridium sensu stricto* 1 exhibiting the highest LDA score of 4.65, which was the highest among those of all identified genera. In terms of relative abundance, *Clostridium sensu stricto* 1 and *Fusobacteriia* are the genera with the most pronounced variations. The cladogram revealed the most correlated branches among groups, which was consistent with the above-mentioned results (**Figure 4B**).

## 4 Discussion

Nowadays, organic acid products are widely used as feed additives to address issues in aquaculture and promote the high-quality development of aquatic products (Dawood et al., 2021; Sotoudeh and Esmaeili, 2022; Abdel-Latif et al., 2020). SB, as an organic acid, has been found to have a significant positive effect on the growth performance of aquatic animals and plays a crucial role in the production of aquatic products (Khalil et al., 2018; Abdel-Latif et al., 2020).

According to the experimental results, dietary SB significantly improved the growth performance of Chinese soft-shelled turtles and promoted feed conversion efficiency, with the strongest effect achieved at an SB concentration of 0.02%. Similar results were also obtained in other studies (da Silva et al., 2016; Abdel-Latif et al., 2021; Mehrgan et al., 2022), all of which showed that adding SB to the diet of aquatic animals could improve their growth efficiency and accelerate their flesh growth.

Studies have found that the long-term consumption of diets containing SB increases intestinal thickness, which in turn increases the contact area between food and the intestinal tract, improves the absorption efficiency of nutrient elements, and promotes the growth of Nile tilapia juveniles (Abdel-Mohsen et al., 2018; Abdel-Tawwab et al., 2021). Additionally, Yamamoto et al. found that disorders in golden drum fish were reversed after adding SB to their feed during the early stages of breeding (Yamamoto et al., 2021). Moreover, Jesus et al. found that SB had little effect on the growth performance of protected and non-protected tilapia (Jesus et al., 2019). This study indicated that there was a dose-benefit relationship between SB and growth performance, which was consistent with the conclusions of Ullah Sami et al. (Ullah et al., 2020) and Zhou et al. (Zhou et al., 2019). Therefore, the efficacy of SB on the growth performance of Chinese soft-shelled turtles depends on the concentration added. When used at an appropriate concentration, SB can greatly improve their growth and feeding efficiency.

Biochemical indexes are important parameters for assessing the welfare of aquatic animals (Mitranescu et al., 2013), and adjusting these indexes can better meet the living conditions of aquatic animals and promote their growth and development. In our study, TC levels in the blood of Chinese soft-shelled turtles fed with SB were lower than those of turtles fed without it, and this was closely related to the increase in SB concentration (Zong et al., 2022). Studies have shown that adding 1–2 mg of SB to the diet of Nile tilapia juveniles can significantly improve the activities of TP and transaminase (Abdel-Latif et al., 2021). Additionally, the TP content in the blood of Nile tilapia was significantly increased when fed a diet containing SB (El-Sayed Ali et al., 2018; Dawood et al., 2020). The concentration of transaminase reflects liver health and function (Kou et al., 2021). Based on the results obtained in this study, there were no significant differences in TP, transaminase, or other indicators between Chinese soft-shelled turtles fed with SB and those in the control group. Furthermore, SB supplementation led to no adverse effects on the original health of Chinese soft-shelled turtles.

Total antioxidant capacity can reflect the enzyme activity in the immune system and inhibit peroxide products *in vivo* (Yarahmadi et al., 2022). SOD can remove free radicals in the body and protect tissues and cells from oxidative interference. Studies have indicated that enzyme activity in yellow catfish was enhanced when fed with 500 or 1000 mg/kg of SB, reaching significantly higher levels than those of the control group (Zhao et al., 2021). However, data on SOD expression in our study differed from these findings. The reason may be that SB has varying effects on different aquatic animals, which requires further research.

Li Tian et al. found that SB could improve complement protein content and enhance immune ability in juvenile grass carp (Tian et al., 2017). As a product of the body’s liver, complement proteins can eliminate inflammation from the body, which has been found that made significant progress in the immunity of aquatic animals in previous studies (Uribe et al., 2011), leading to the rapid development of the aquatic industry. In this experiment, feed supplemented with SB resulted in a significant enhancement in the levels of complement proteins, which aligns with the findings mentioned in the literature. This indicated that the addition of SB to the feed can enhance the immune system of Chinese soft-shelled turtles.

The results of our experiment showed that feeding diets containing SB had a positive effect on the beneficial intestinal microflora of Chinese soft-shelled turtles, which is consistent with the findings of Wing‐Keong Ng et al. (Ng and Koh, 2017). It has been reported that SB can increase intestinal acidity and inhibit the abundance of harmful microbial populations (Ricke, 2003). However, the mechanism by which SB can promote changes in intestinal microbial flora is not fully understood (Dibner and Buttin, 2002). It is generally believed that SB can form a physical barrier in the intestine to protect the intestinal microbial flora from damage (Wu et al., 2018).

In this experiment, adding 0.02% SB to the diet significantly improved the intestinal tract of Chinese soft-shelled turtles, enhanced the abundance of beneficial microbial populations in the intestine, and promoted intestinal digestion and absorption, which is similar to the conclusion of Han et al. (Han et al., 2020). Briefly, at the phylum level, *Proteobacteria*, *Firmicutes*, and *Bacteroidota* were found to be the dominant phyla. At the genus level, *Citrobacter* and *Bacteroides* were found to be the main dominant bacteria. *Polysaccharides* in the gut require specific digestive enzymes to decompose them due to their different structure compared to other foods (Porter and Martens, 2017). When these digestive enzymes are lacking in the intestine, bacteria in this organ must decompose them for absorption (Ai et al., 2019). In Chinese soft-shelled turtles, *Bacteroides* has been found to be predominant in the intestinal tract, which can digest dietary fiber *polysaccharides* and main carbohydrates (Porter et al., 2018), promoting the digestion and absorption of carbohydrates in the intestine and overall growth. This study found that the abundance of *Bacteroides* species in the intestinal tract of Chinese soft-shelled turtles increased, which improved the efficiency of feed digestion and utilization and accelerated the growth of Chinese soft-shelled turtles supplemented with an SB diet. Kan et al. (Kan et al., 2015), found that when the external environment changed, the microbial flora in the gut of crucian carp would be adjusted in a beneficial direction.

This study showed that SB had a beneficial effect on the intestinal tract of Chinese soft-shelled turtles, improving their growth performance, blood indexes, and intestinal microbiota compared to those in the control group. When the indexes changed, other influencing factors also changed. However, our exploration of the mechanism of the influence of SB on intestinal microorganisms is limited, and further research is needed to provide a reference for the subsequent establishment of an SB dietary supplementation system for Chinese soft-shelled turtles.

## 5 Conclusion

In conclusion, 0.02% SB supplementation could improve the growth performance, feed digestibility, and gut microbiota of Chinese soft-shelled turtles, promoting their healthy growth. In addition, this study found that SB improved the serum biochemical indexes and immune ability of Chinese soft-shelled turtles, including the activity of blood complement proteins. Subsequent trials should continue to investigate SB as a diet supplement for Chinese soft-shelled turtles and explore the actual dosage range of SB.

## Data availability statement

The data presented in the study are deposited in the SequenceRead Archive (https://www.ncbi.nlm.nih.gov/sra), under accession number PRJNA1000466 (https://www.ncbi.nlm.nih.gov/bioproject/PRJNA1000466/).

## Ethics statement

The animal study was approved by College of Fisheries, Hunan Agriculture University. The study was conducted in accordance with the local legislation and institutional requirements.

## Author contributions

XW, ZD, and YH conceived the study. LG, YY, and KL performed the experiments. LG, HX, and QL analyzed the data. LG, YY, and SL visualized the statistical results. LG wrote the draft. XW, and YH wrote and edited the final manuscript. ZL improved the language and grammar of the manuscript. All authors contributed to the article and approved the submitted version.
